# Supplementary material for: Mystery of the Passerini Reaction for the Synthesis of the Antimicrobial Peptidomimetics against Nosocomial Pathogenic Bacteria
Source: Int J Mol Sci. 2024 Jul 30;25(15):8330. doi: 10.3390/ijms25158330 (PMC11312933; doi:10.3390/ijms25158330)
Supplement: Supplementary file 1 [file ijms-25-08330-s001.zip › ijms-3085424-supplementary.pdf]

Supporting Information

Mystery of the Passerini Reaction for the Synthesis of the Antimicrobial Peptidomimetics against Nosocomial Pathogenic Bacteria

Deepak S. Wavhal [1], Dominik Koszelewski [1], Cezary Gulko [1], Paweł Kowalczyk [2,*], Anna Brodzka [1], Karol Kramkowski [3] and Ryszard Ostaszewski [1,*]

[1] Institute of Organic Chemistry, Polish Academy of Sciences, Kasprzaka 44/52, 01-224 Warsaw, Poland; deepak.wavhal@icho.edu.pl (D.S.W.); dominik.koszelewski@icho.edu.pl (D.K.); cezary.gulko@icho.edu.pl (C.G.); anna.brodzka@icho.edu.pl (A.B.)

[2] Department of Animal Nutrition, The Kielanowski Institute of Animal Physiology and Nutrition, Polish Academy of Sciences, Instytucka 3, 05-110 Jabłonna, Poland

[3] Department of Physical Chemistry, Medical University of Bialystok, Kilińskiego 1 Str., 15-089 Białystok, Poland; kkramk@wp.pl

* Correspondence: p.kowalczyk@ifzz.pl (P.K.); ryszard.ostaszewski@icho.edu.pl (R.O.); Tel.: +48-227653301 (P.K.); +48-223432054 (R.O.)

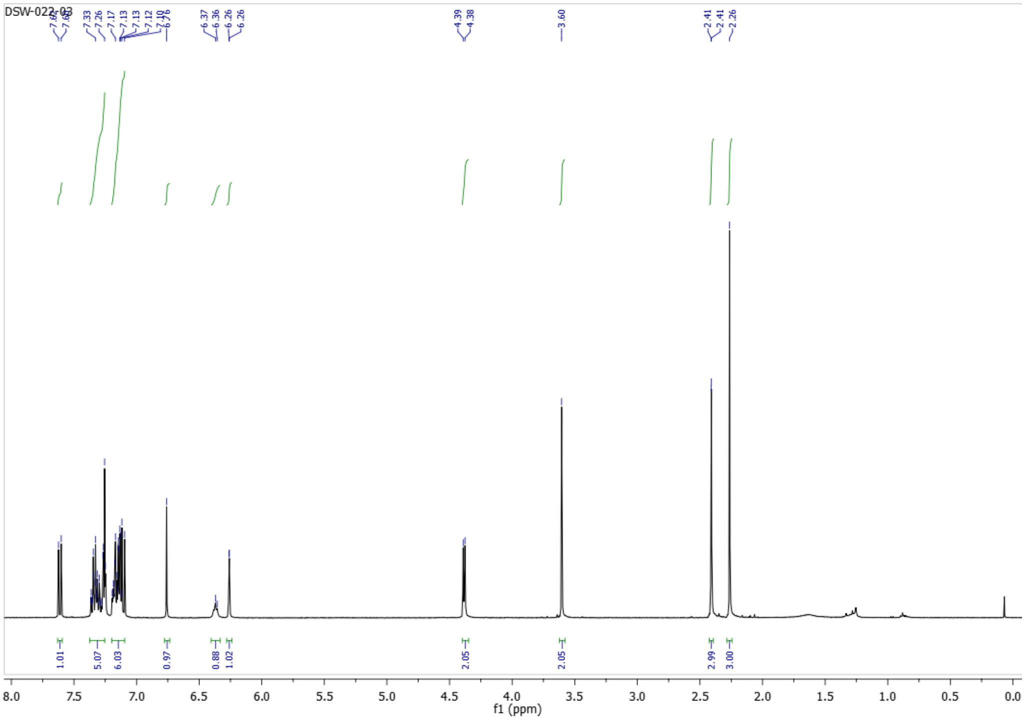

Figure S1. 1H NMR (400 MHz, CDCl3) spectra of compound **1**

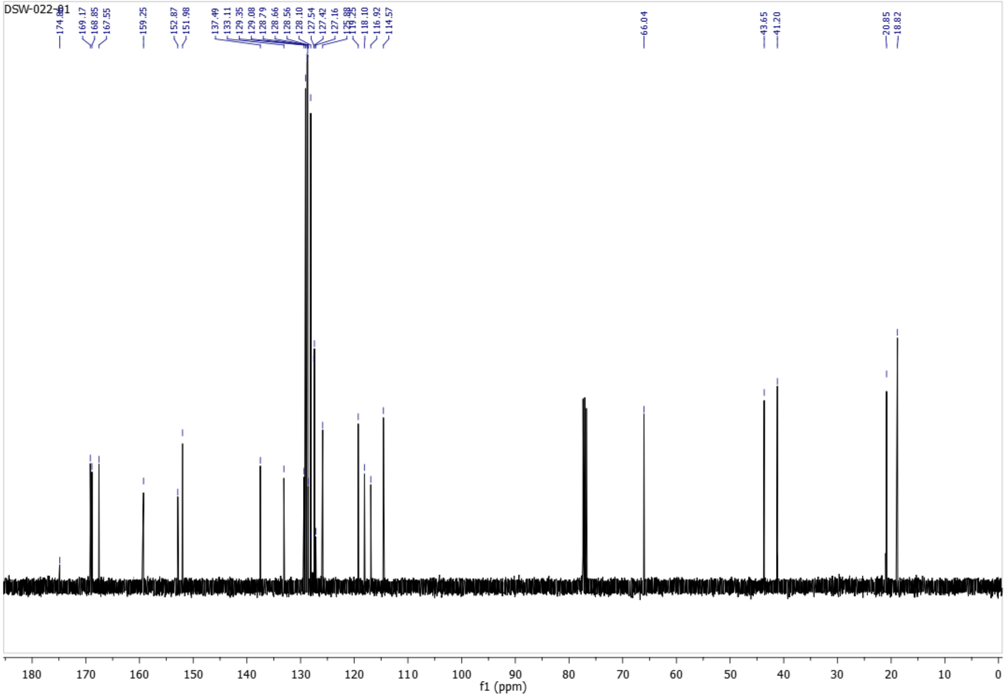

Figure S2. ^{13}C NMR (100 MHz, $CDCl_3$) spectra of compound **1**

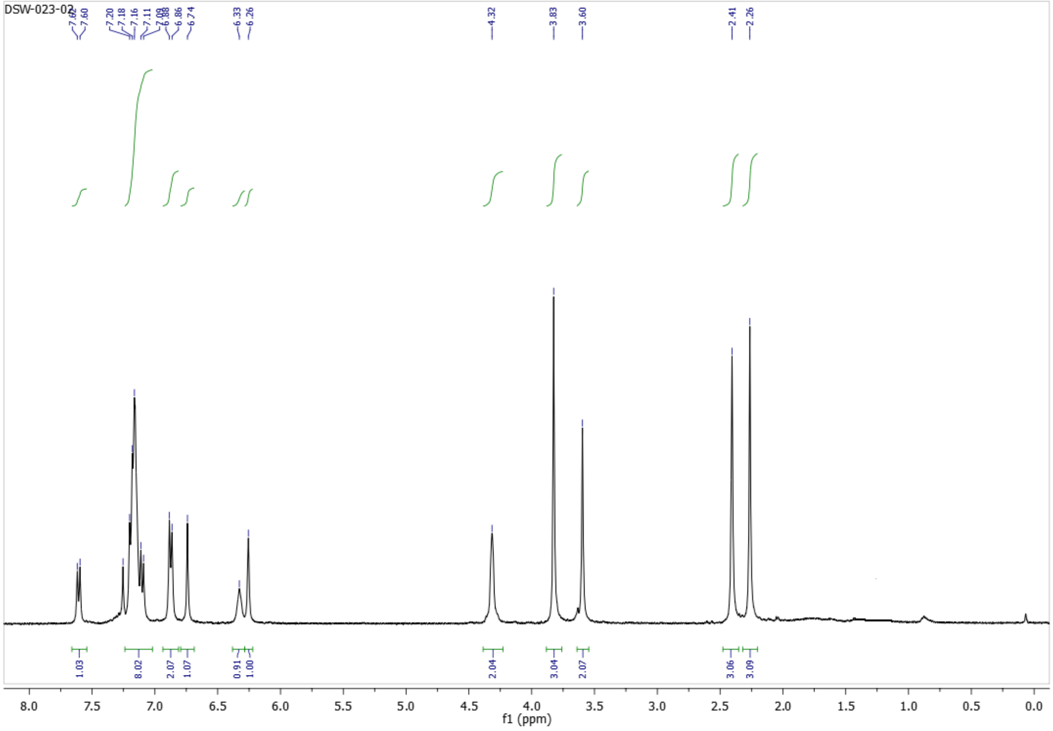

Figure S3. ^{1}H NMR (400 MHz, $CDCl_3$) spectra of compound **2**

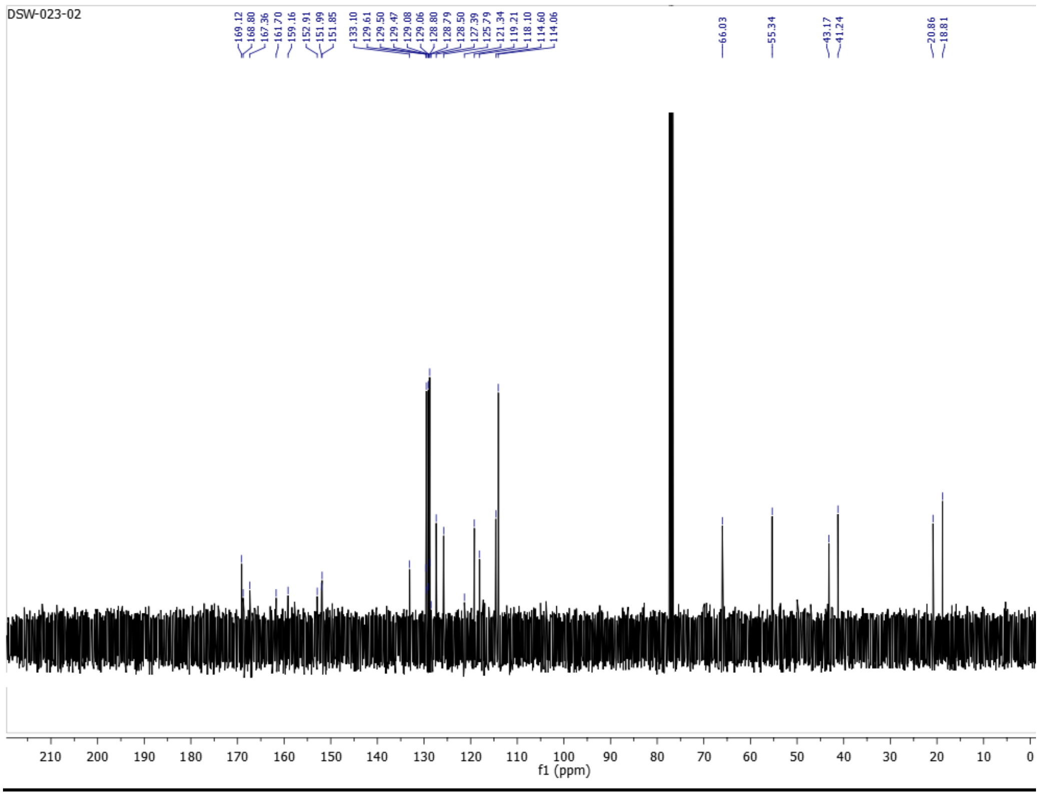

Figure S4. ^{13}C NMR (100 MHz, CDCl**3**) spectra of compound **2**

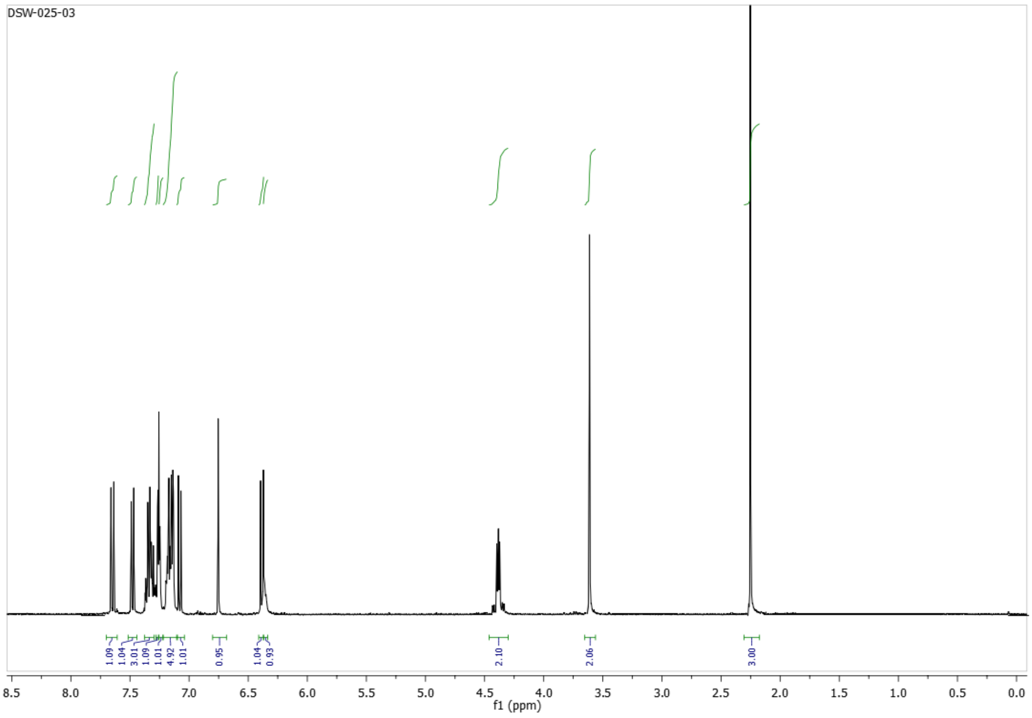

Figure S5. ^{1}H NMR (400 MHz, $CDCl_3$) spectra of compound **3**

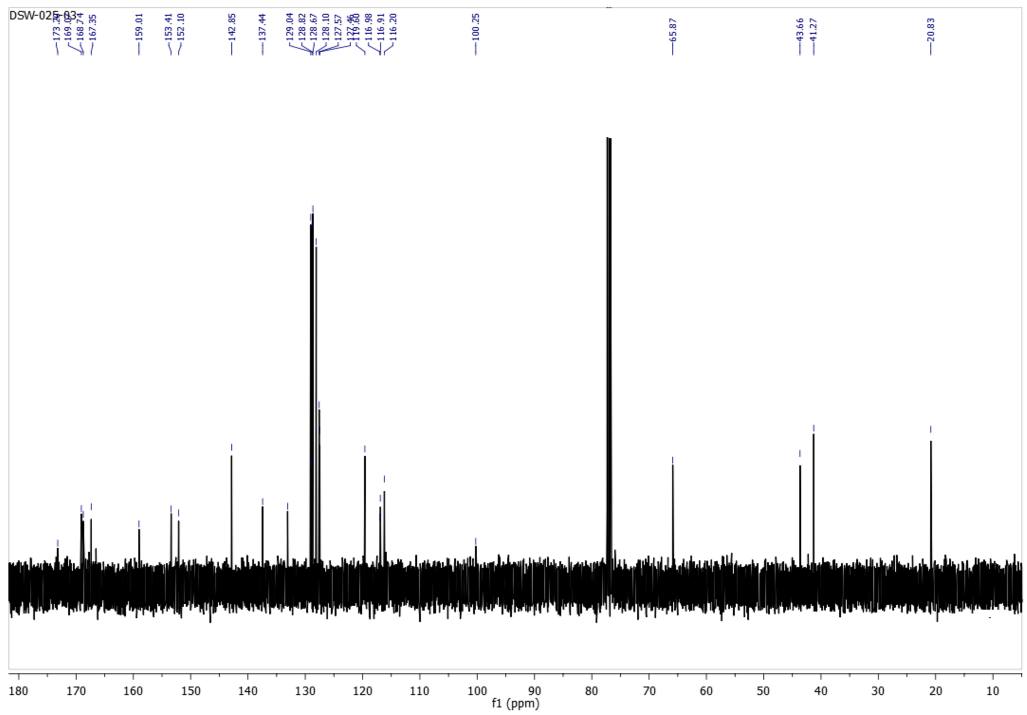

Figure S6. ^{13}C NMR (100 MHz, $CDCl_3$) spectra of compound **3**

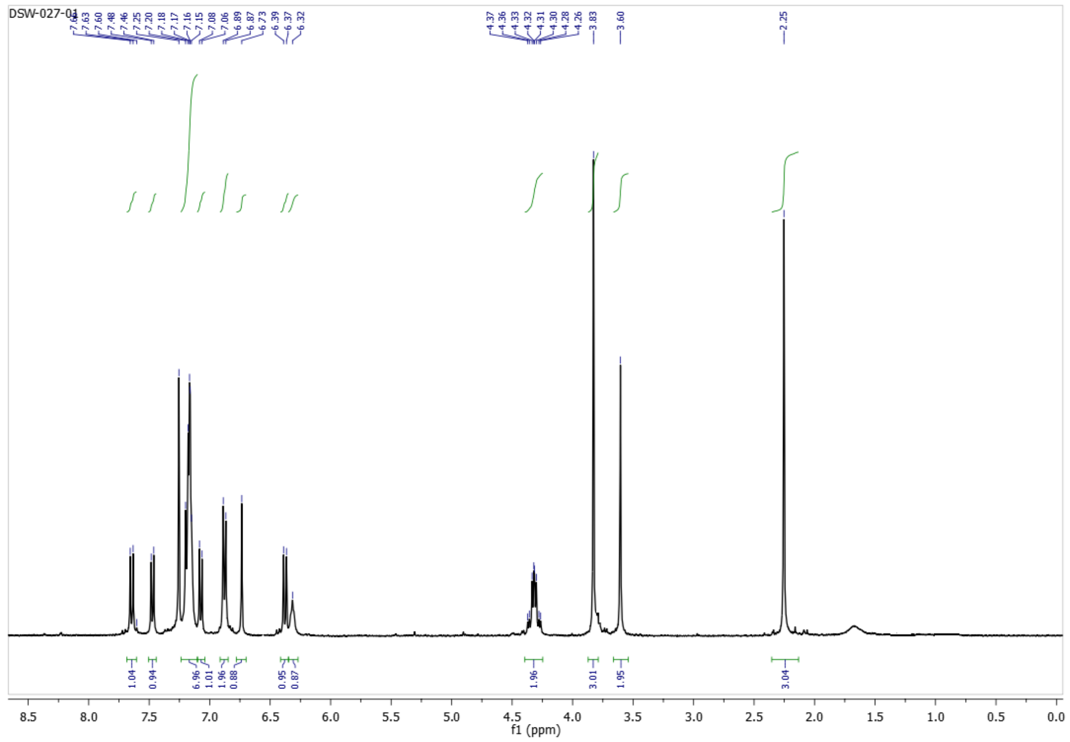

Figure S7. ^{1}H NMR (400 MHz, $CDCl_3$) spectra of compound **4**

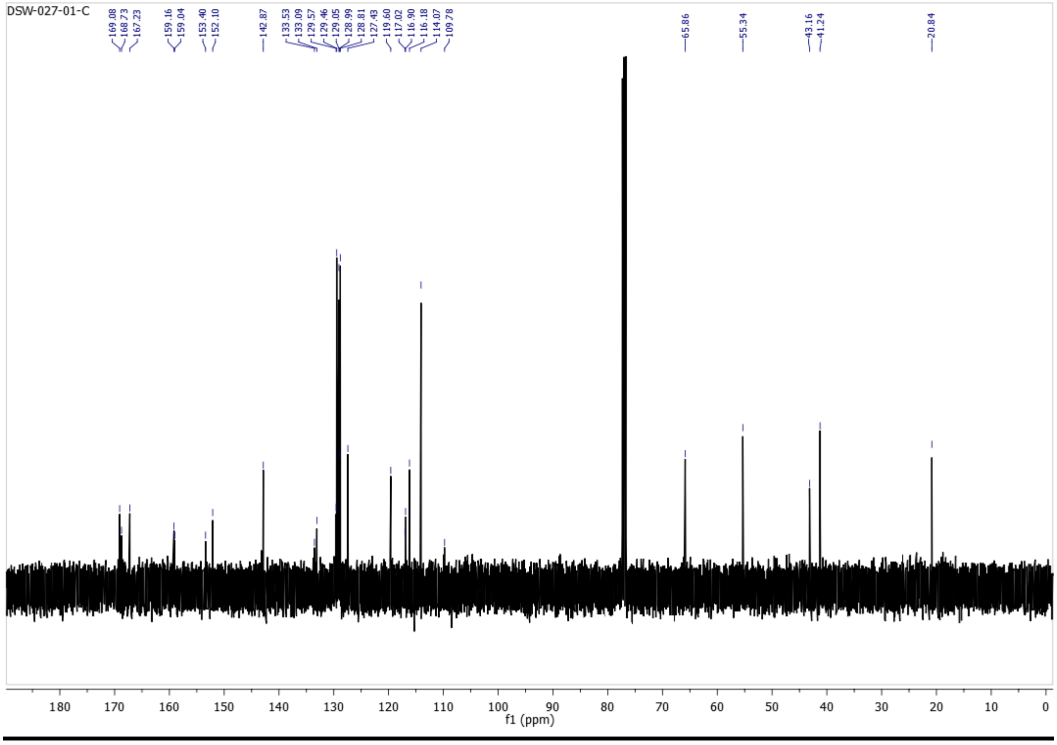

Figure S8. ^{13}C NMR (100 MHz, $CDCl_3$) spectra of compound **4**

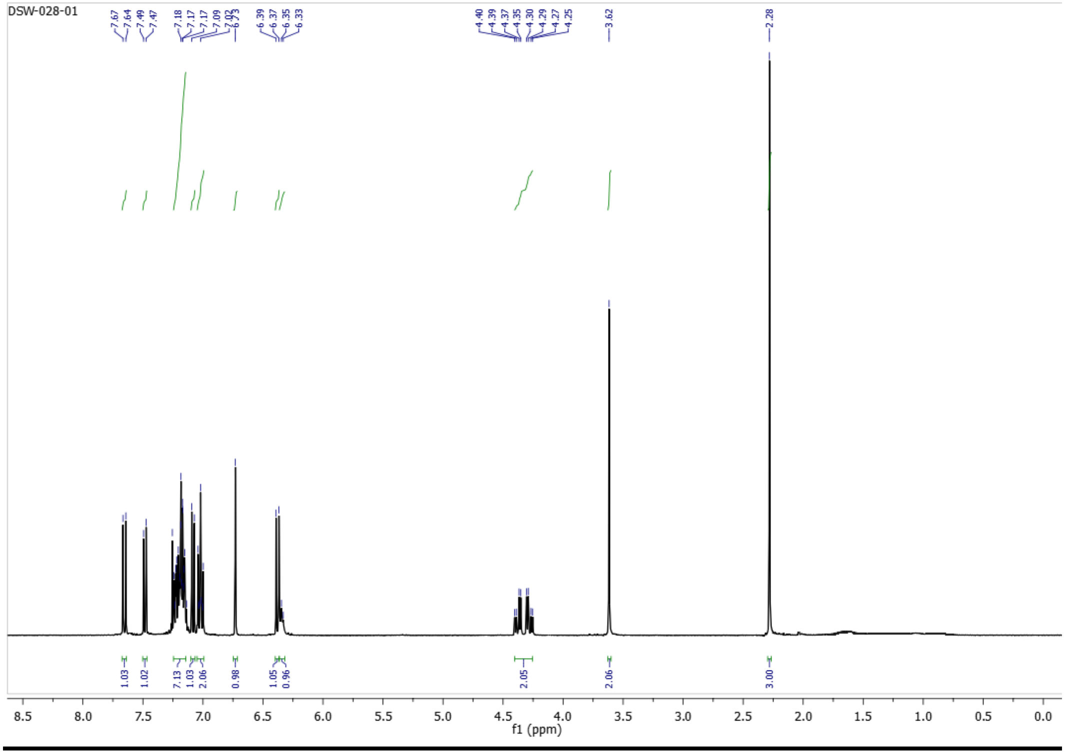

Figure S9. ^{1}H NMR (400 MHz, $CDCl_3$) spectra of compound **5**

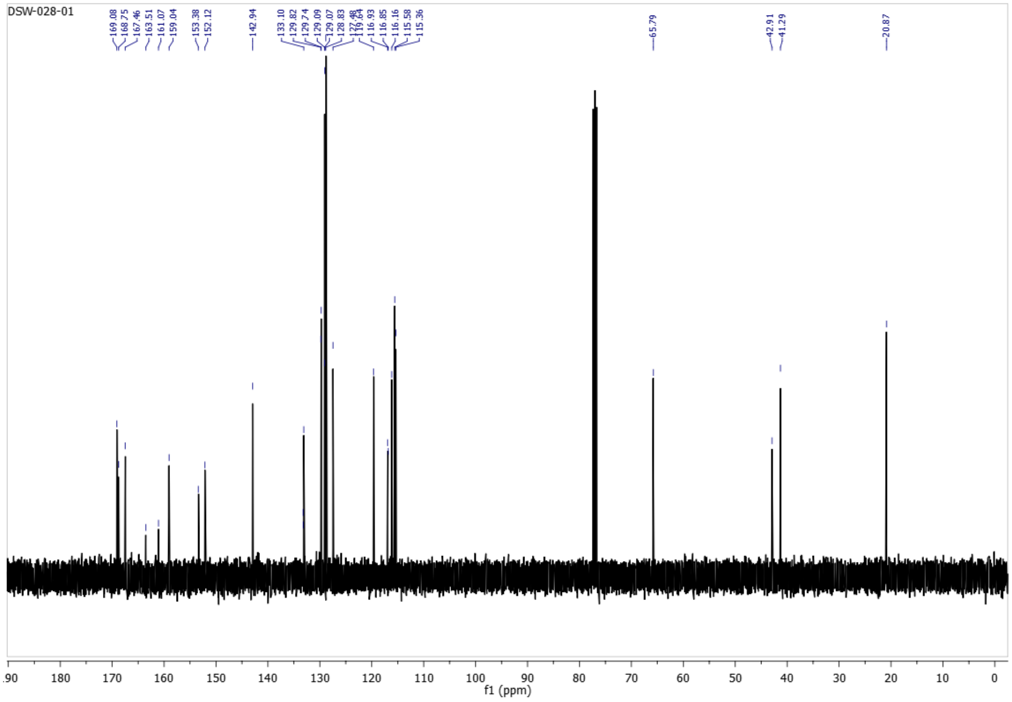

Figure S10. ^{13}C NMR (100 MHz, $CDCl_3$) spectra of compound **5**

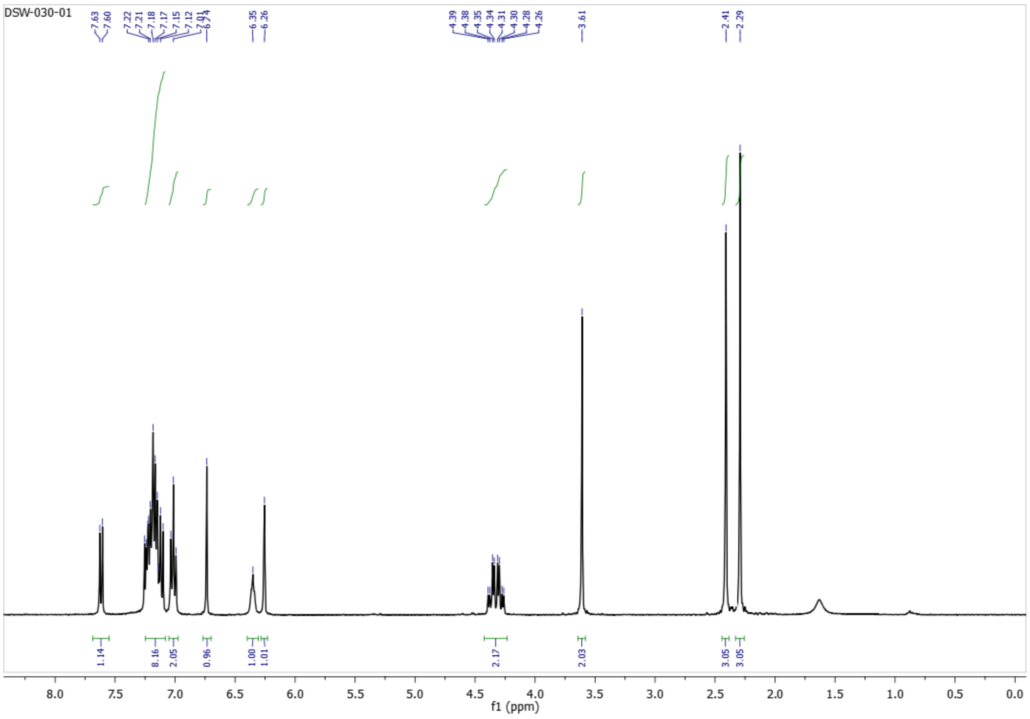

Figure S11. ^{1}H NMR (400 MHz, $CDCl_3$) spectra of compound **6**

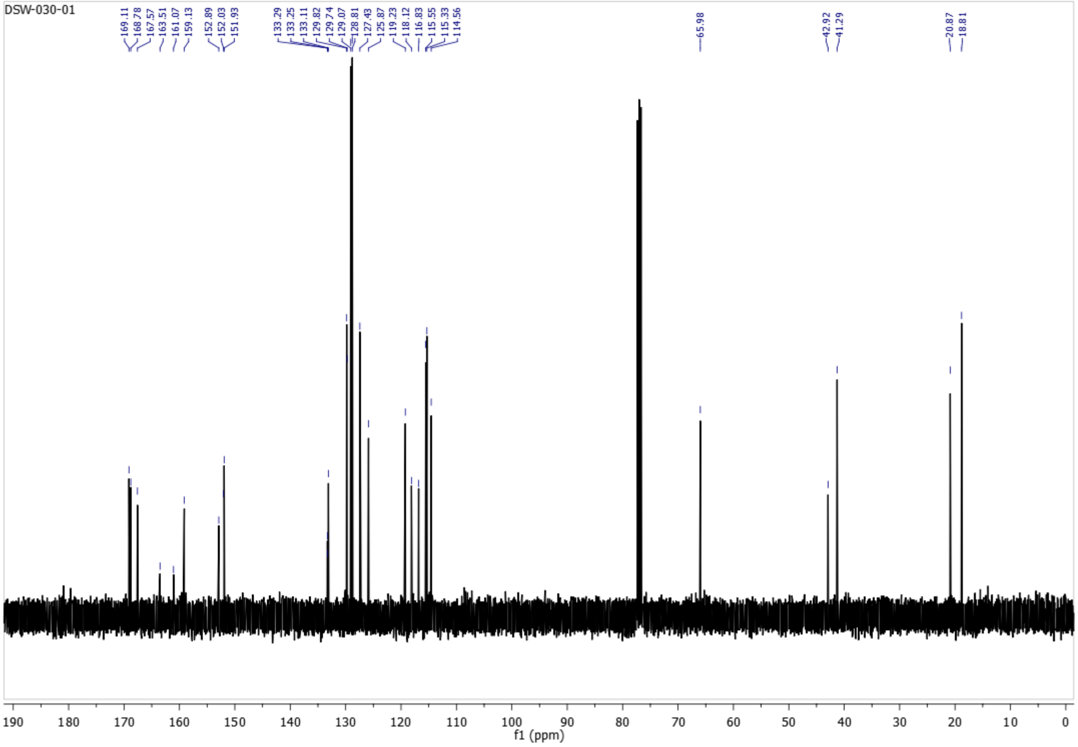

Figure S12. ^{13}C NMR (100 MHz, $CDCl_3$) spectra of compound **6**

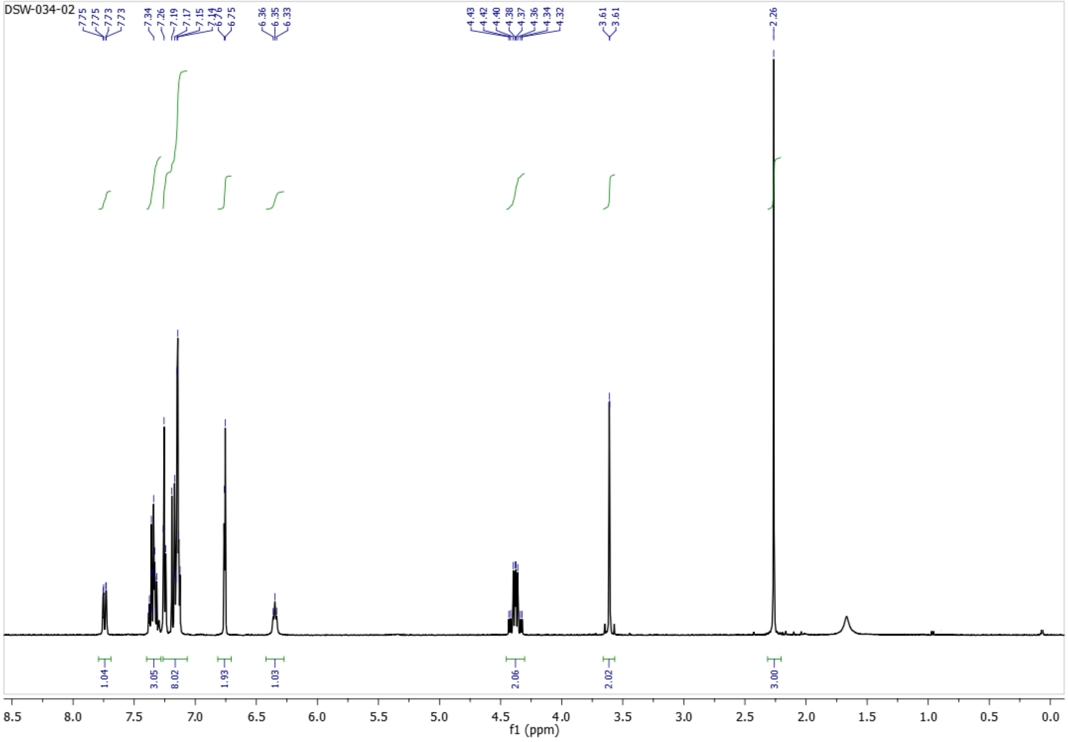

Figure S13. ^{1}H NMR (400 MHz, $CDCl_3$) spectra of compound **7**

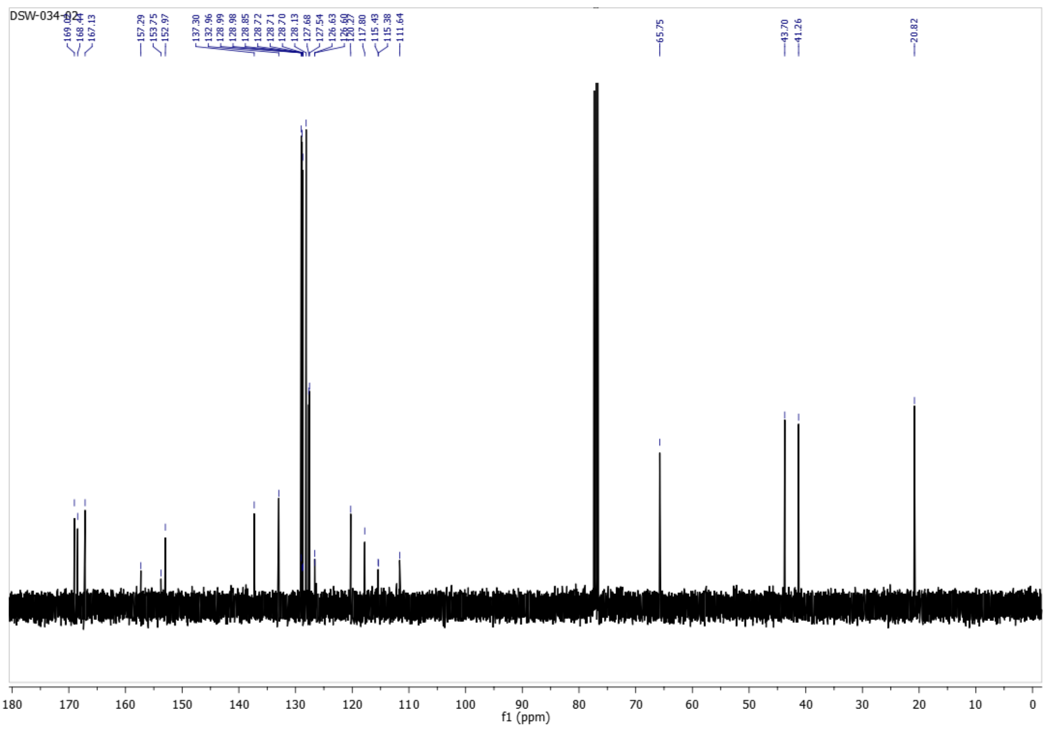

Figure S14. ^{13}C NMR (400 MHz, $CDCl_3$) spectra of compound **7**

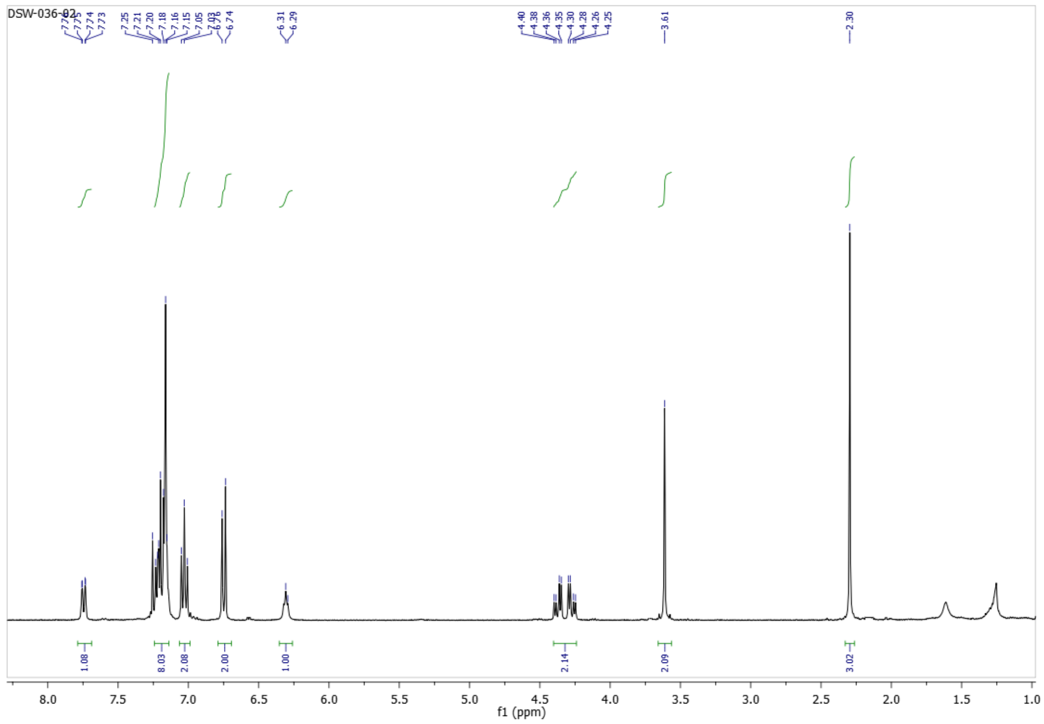

Figure S15. ^{1}H NMR (400 MHz, $CDCl_3$) spectra of compound **8**

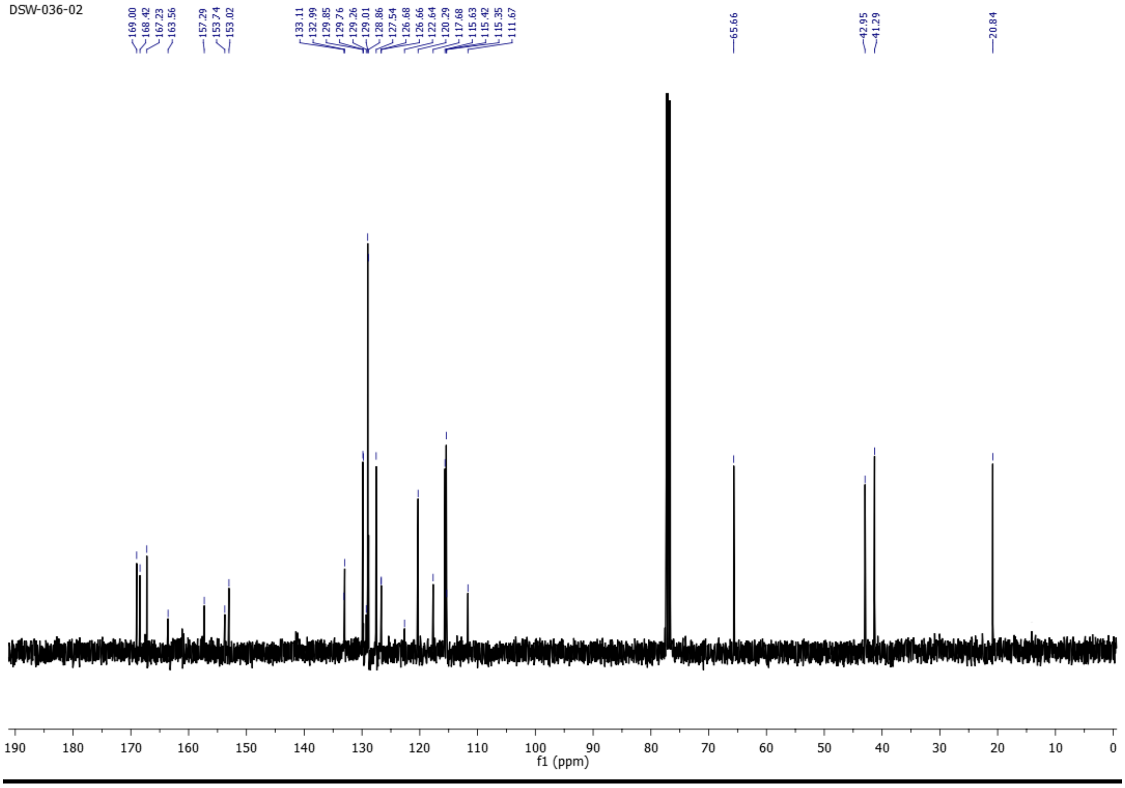

Figure S16. ^{13}C NMR (100 MHz, $CDCl_3$) spectra of compound **8**

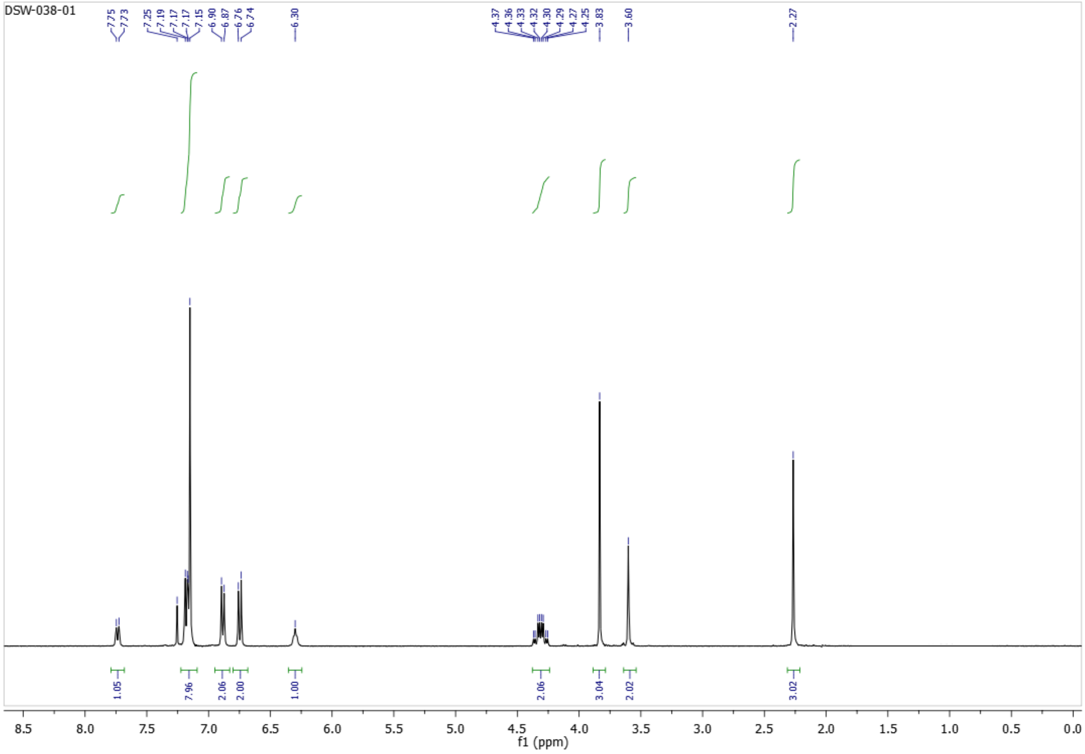

Figure S17. ^{1}H NMR (400 MHz, $CDCl_3$) spectra of compound **9**

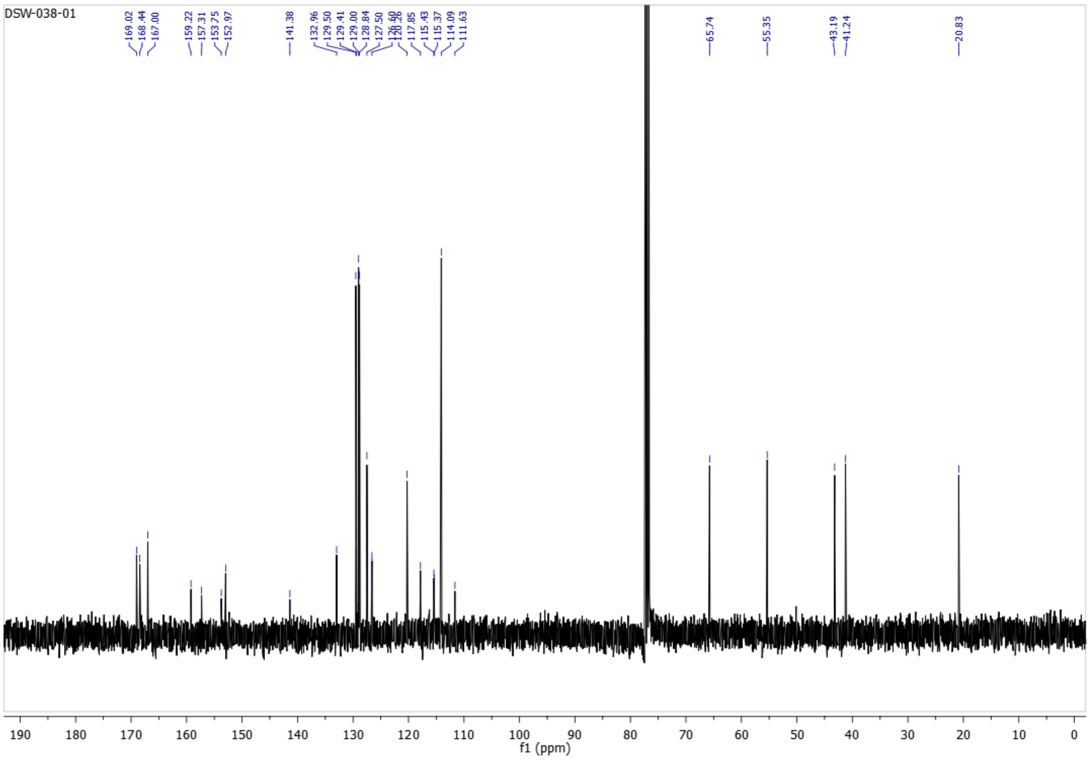

Figure S18. ^{13}C NMR (100 MHz, $CDCl_3$) spectra of compound **9**

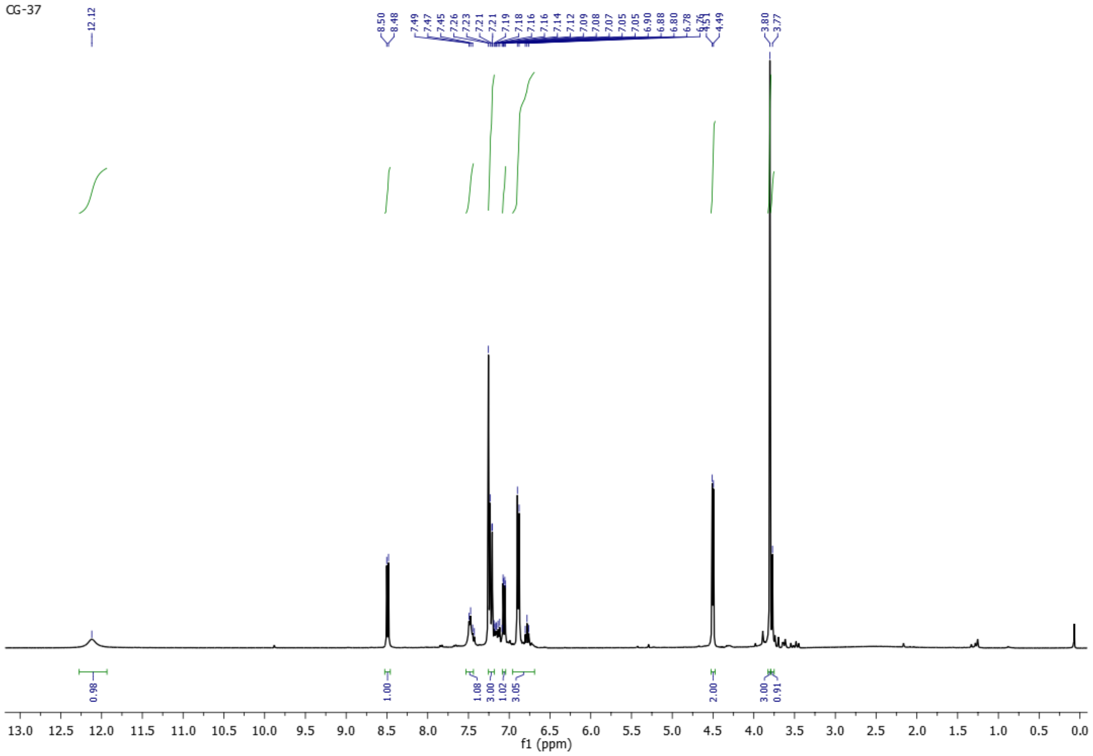

Figure S19. ^{1}H NMR (400 MHz, $CDCl_3$) spectra of compound **10**

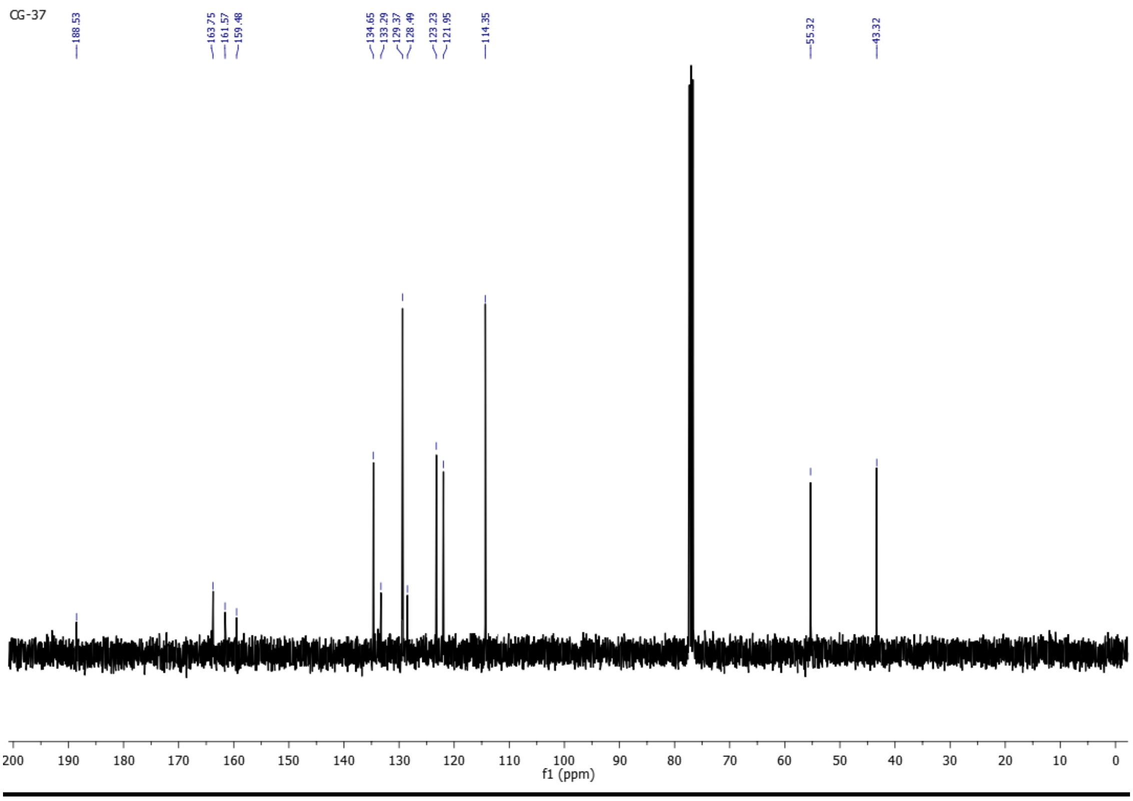

Figure S20. ^{13}C NMR (100 MHz, $CDCl_3$) spectra of compound **10**

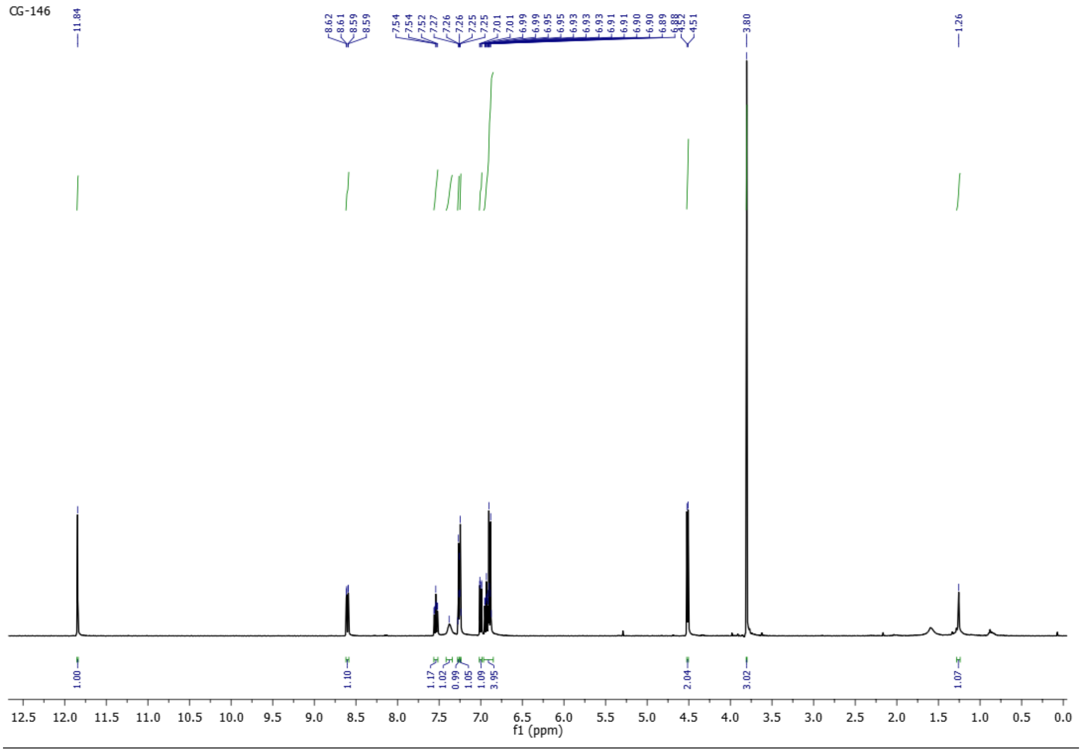

Figure S21. ^{1}H NMR (400 MHz, $CDCl_3$) spectra of compound **11**

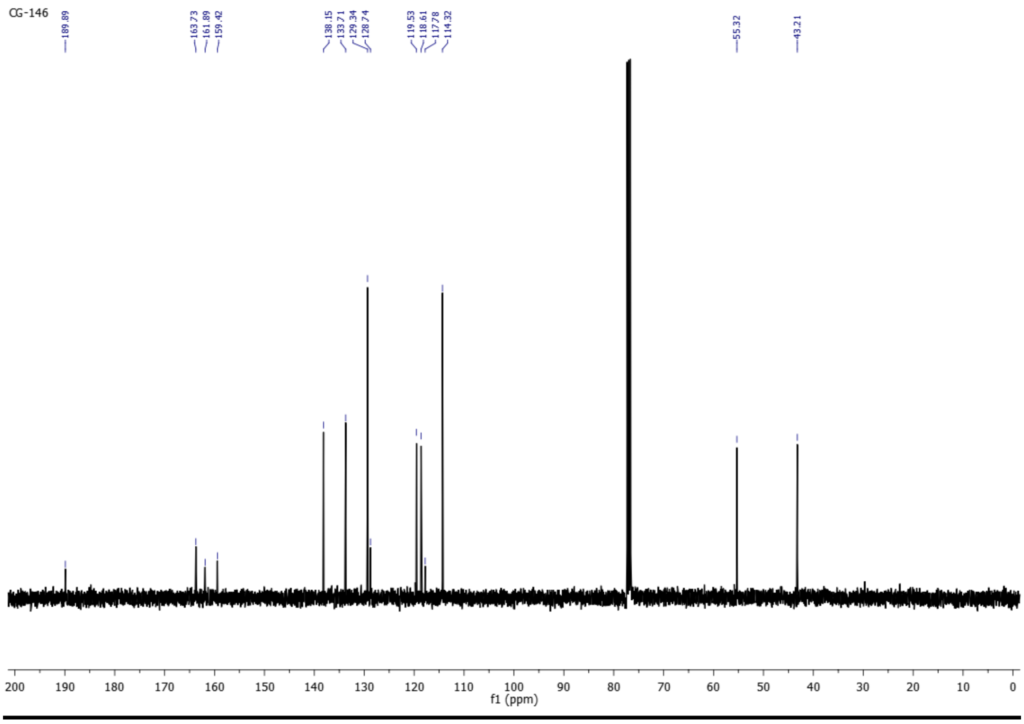

Figure S22. ^{13}C CMR (100 MHz, $CDCl_3$) spectra of compound **11**

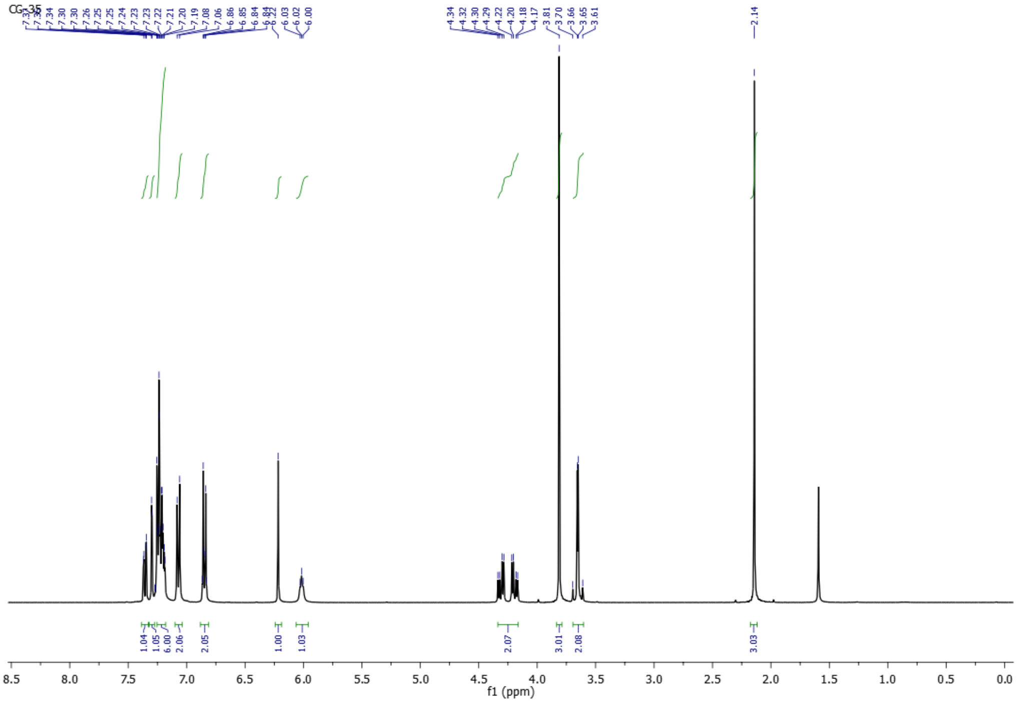

Figure S23. ^{1}H NMR (400 MHz, $CDCl_3$) spectra of compound **12**

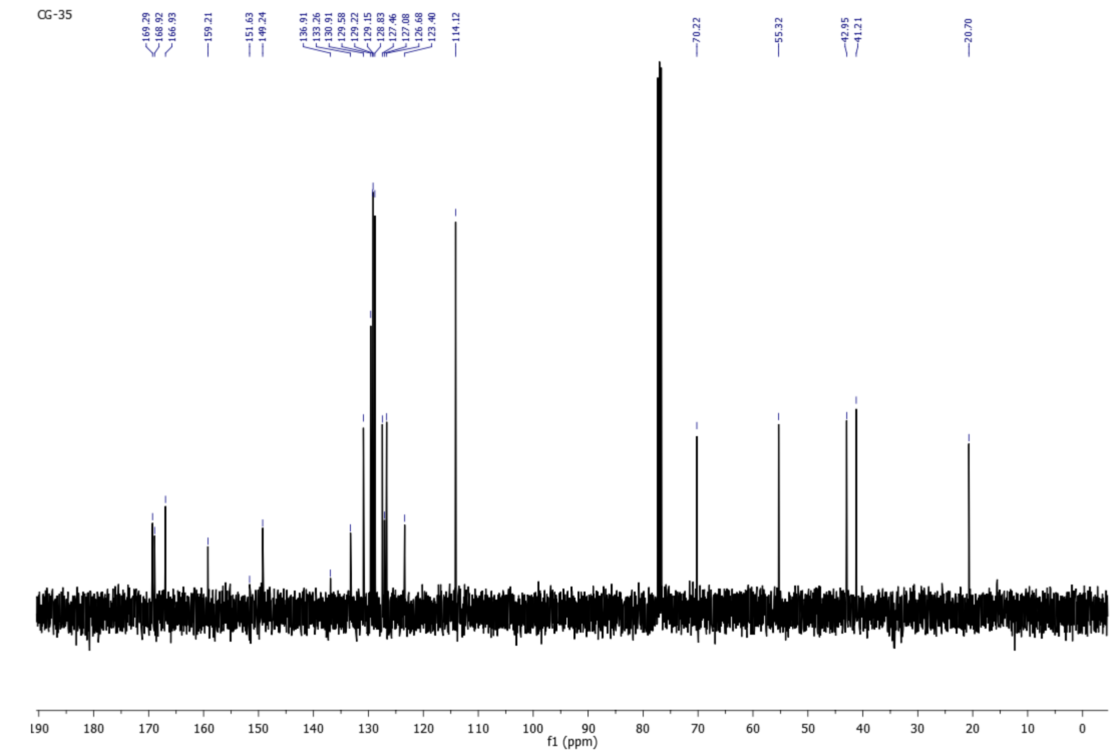

Figure S24. ^{13}C NMR (100 MHz, $CDCl_3$) spectra of compound **12**

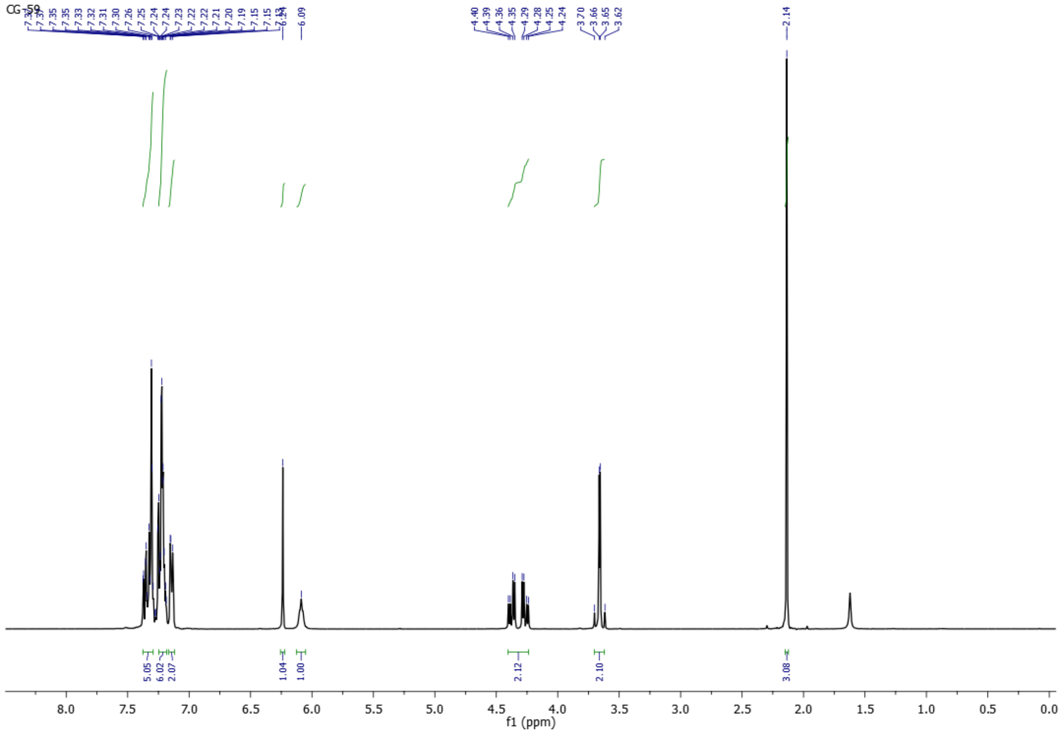

Figure S25. ^{1}H NMR (400 MHz, $CDCl_3$) spectra of compound **13**

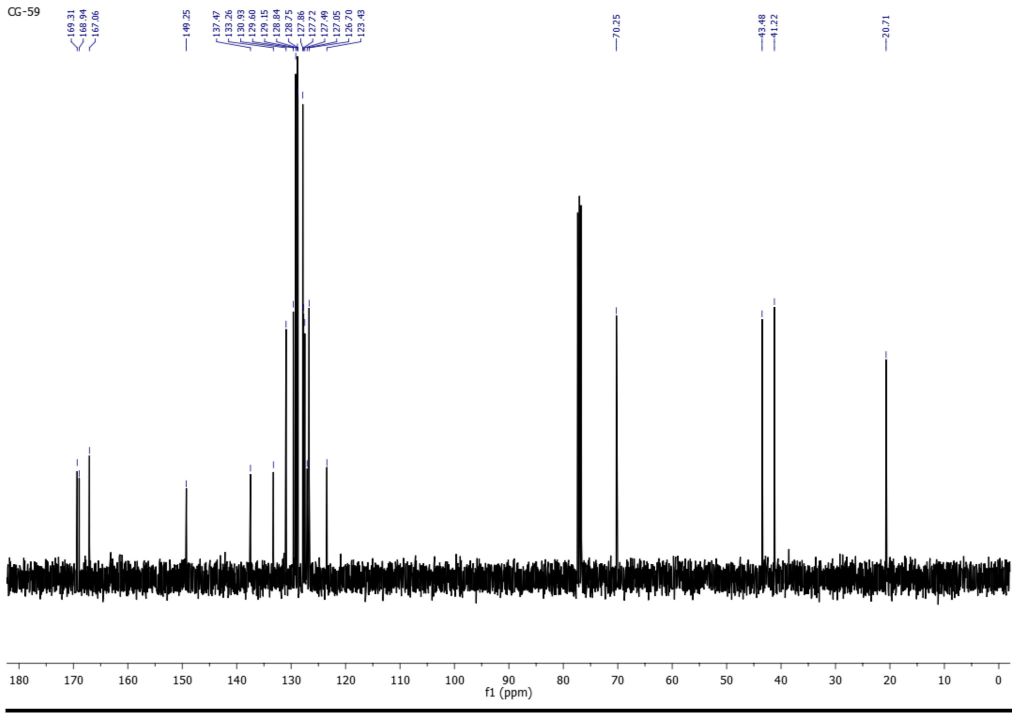

Figure S26. ^{13}C NMR (100 MHz, $CDCl_3$) spectra of compound **13**

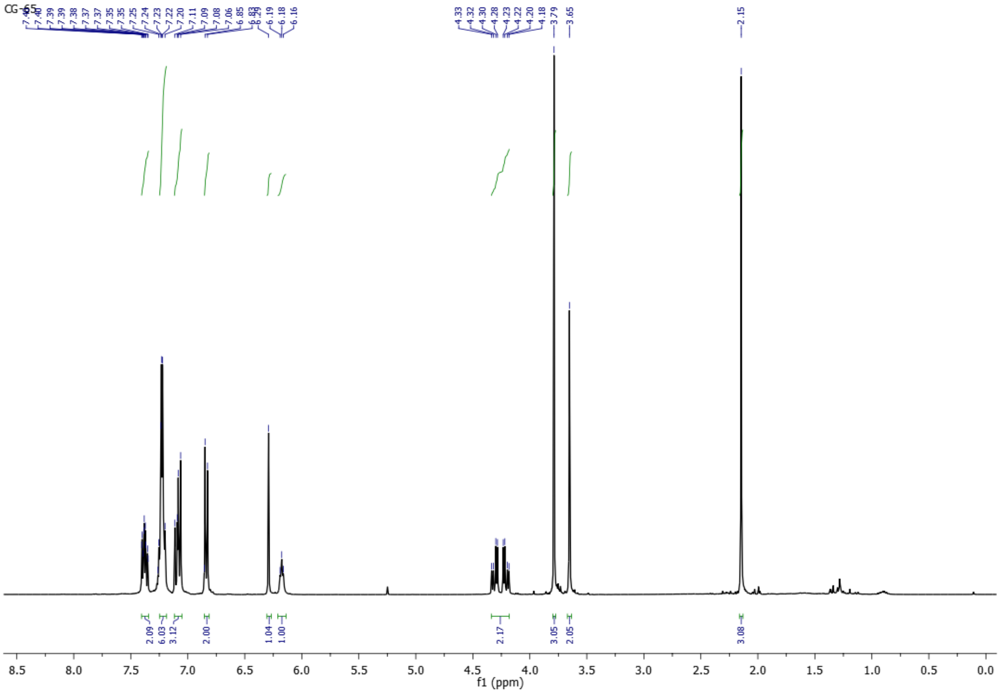

Figure S27. ^{1}H NMR (400 MHz, $CDCl_3$) spectra of compound **14**

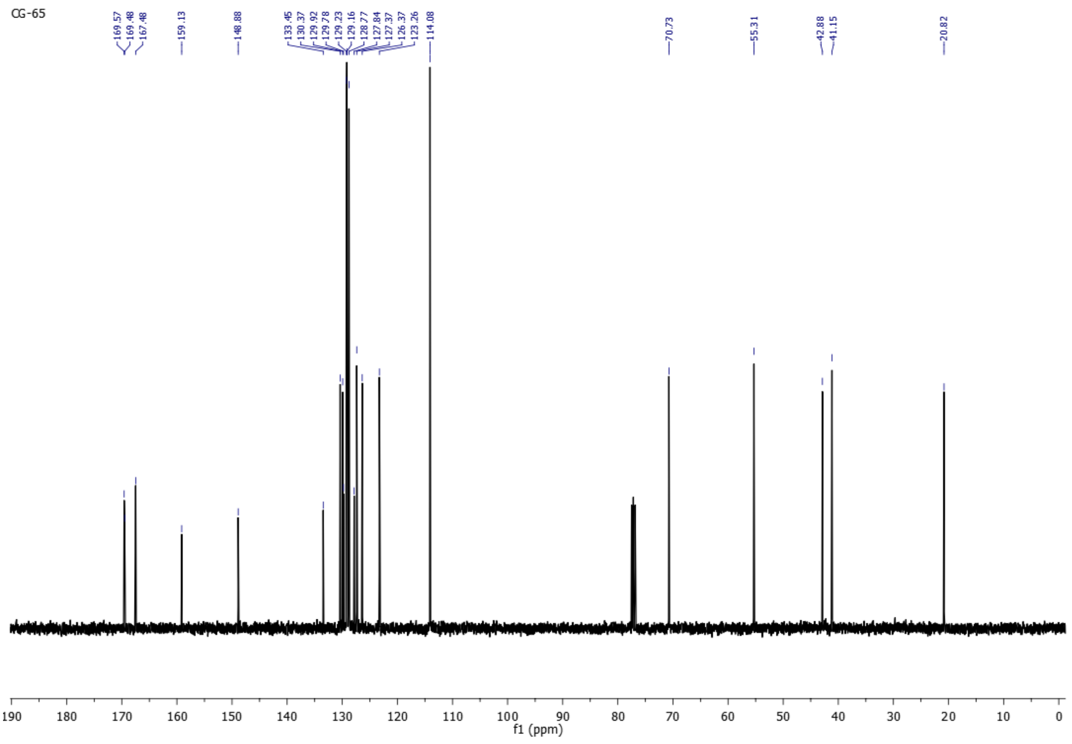

Figure S28. ^{13}C NMR (100 MHz, $CDCl_3$) spectra of compound **14**

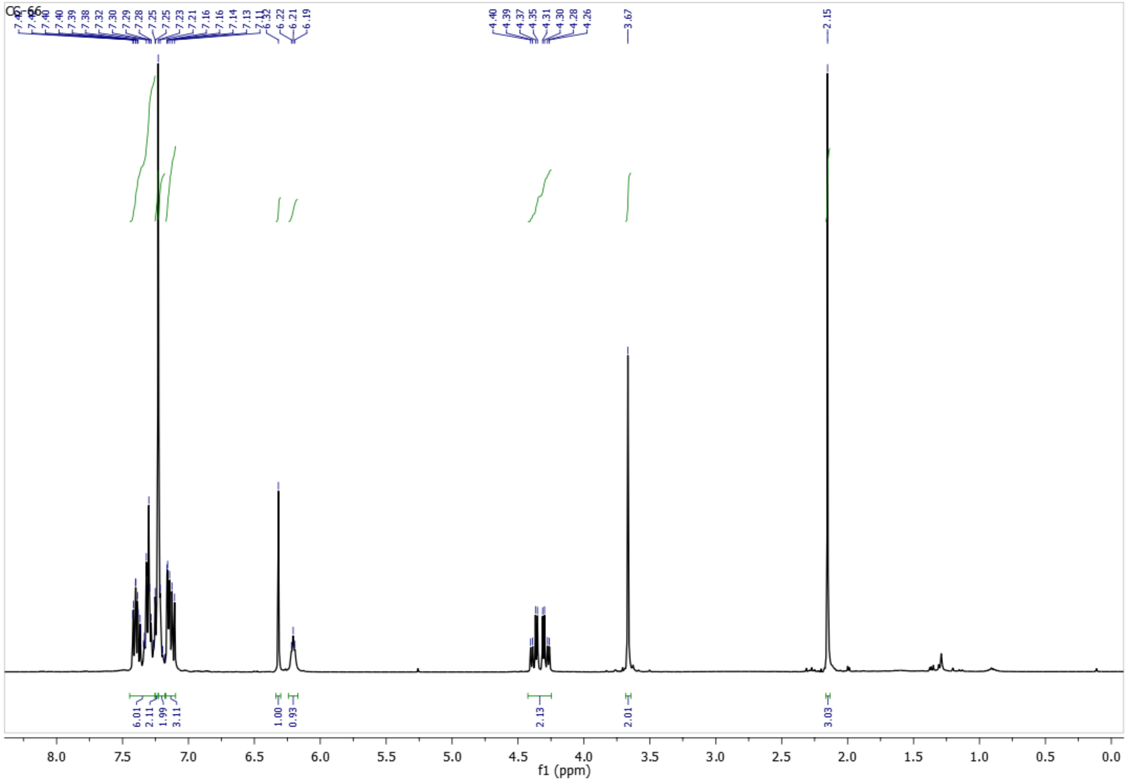

Figure S29. ^{1}H NMR (400 MHz, $CDCl_3$) spectra of compound **15**

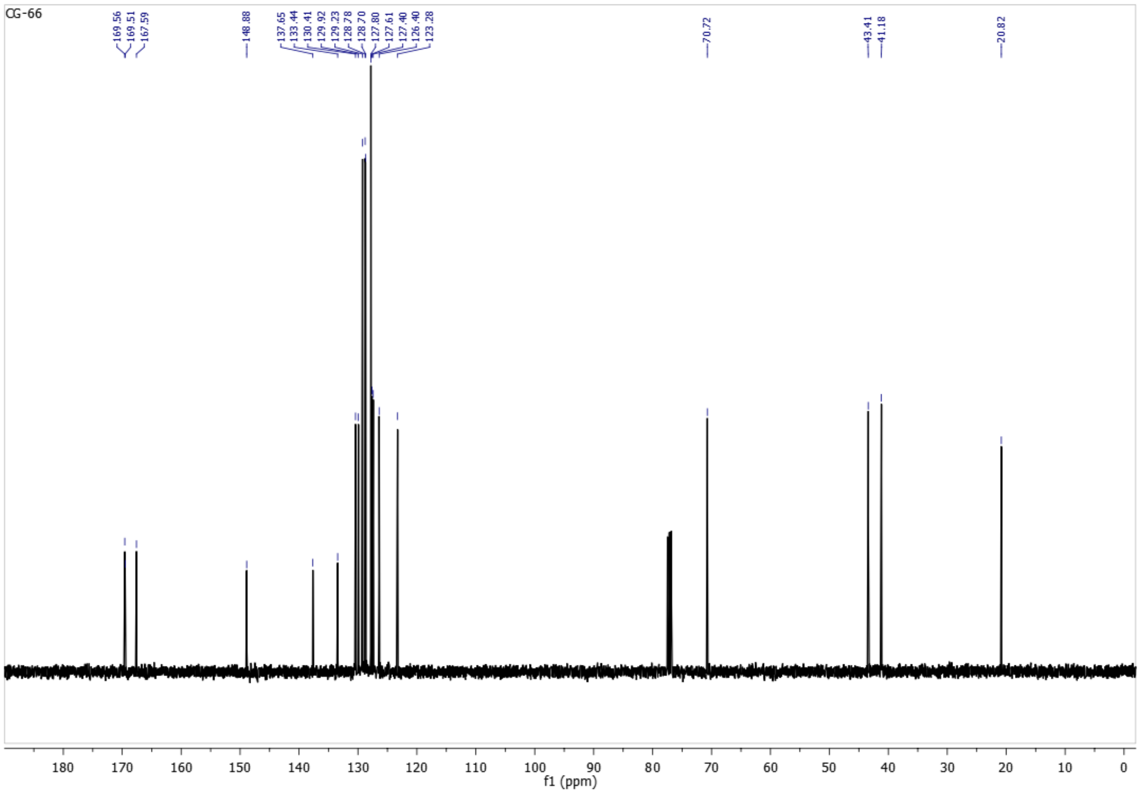

Figure S30. ^{13}C NMR (100 MHz, $CDCl_3$) spectra of compound **15**

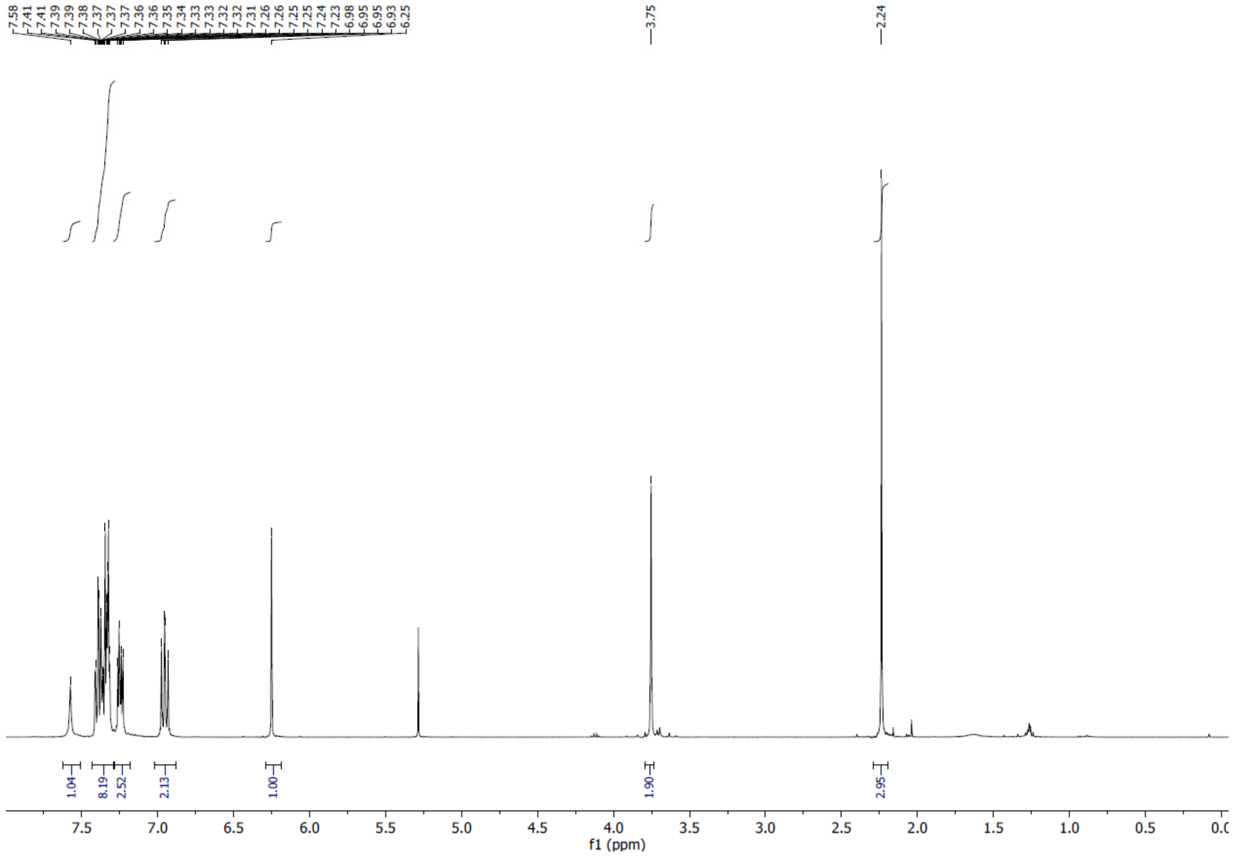

Figure S31. ^{1}H NMR (400 MHz, $CDCl_3$) spectra of compound **16**

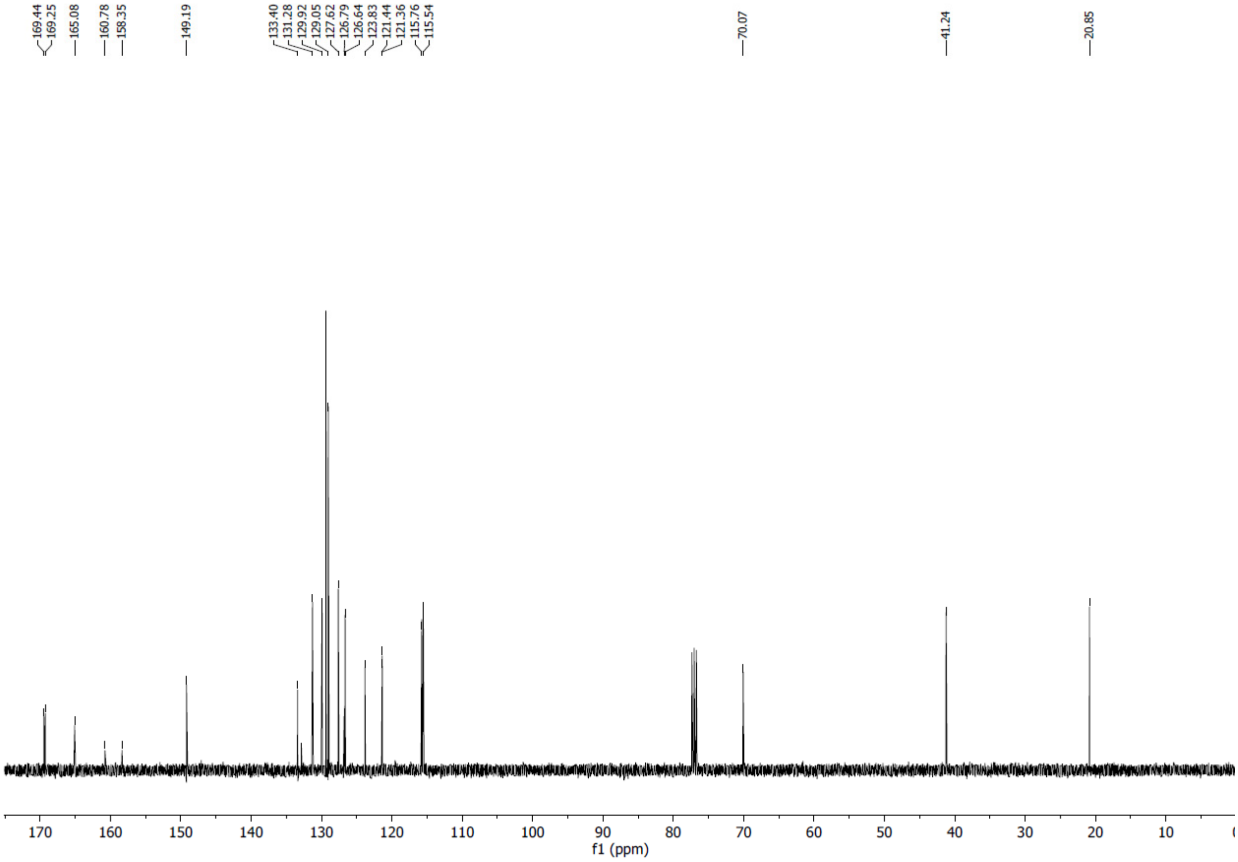

Figure S32. ^{13}C NMR (100 MHz, $CDCl_3$) spectra of compound **16**

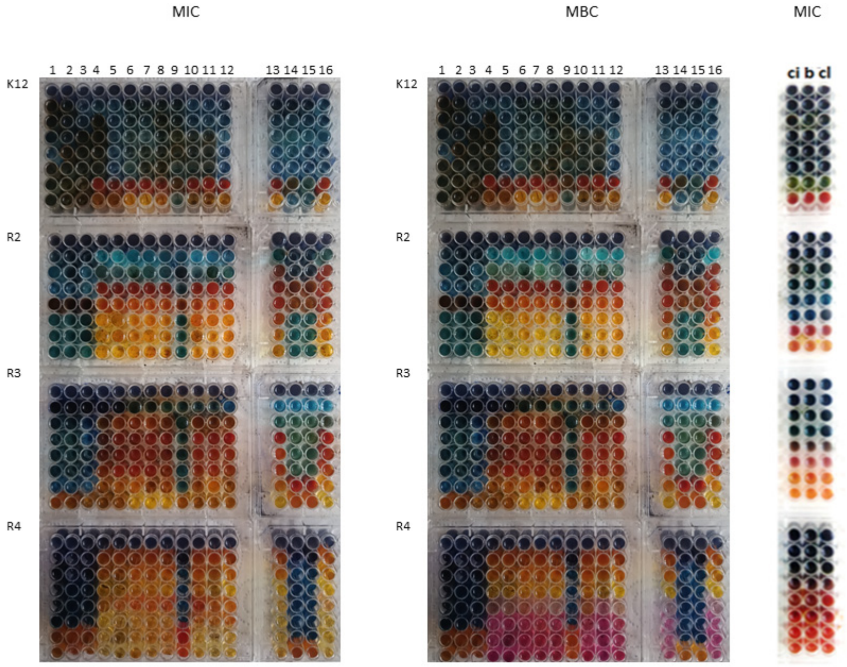

Figure S33. Examples of MIC and MBC on microplates with different concentration of studied compounds (μg/mL^{-1}). Resazurin was added as an indicator of microbial growth with K12, R2, R3, and R4 strains with tested compounds, as described in Figure 3 and 4. Additionally, examples of MIC with different strains K12, R2, R3, and R4 of studied antibiotics with ciprofloxacin (ci), bleomycin (b), and cloxacillin (cl) in (μg/mL^{-1}).

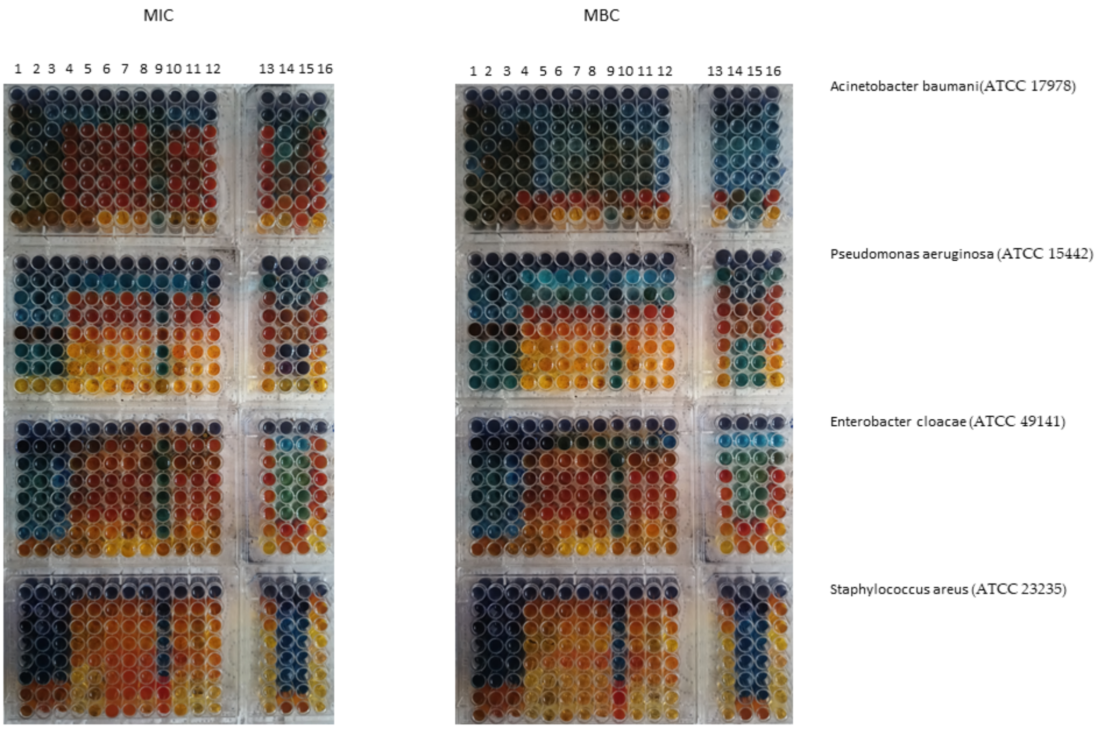

Figure S34. Examples of MIC and MBC on microplates with different concentration of studied compounds (μg/mL^{-1}). Resazurin was added as an indicator of microbial growth with *Staphylococcus aureus strain* (ATCC 23235), as well as on *Acinetobacter baumannii* (ATCC 17978), *Pseudomonas aeruginosa* (ATCC 15442), *Enterobacter cloacae* (ATCC 49141) strains with tested compounds, as described in Figure 3 and 4.

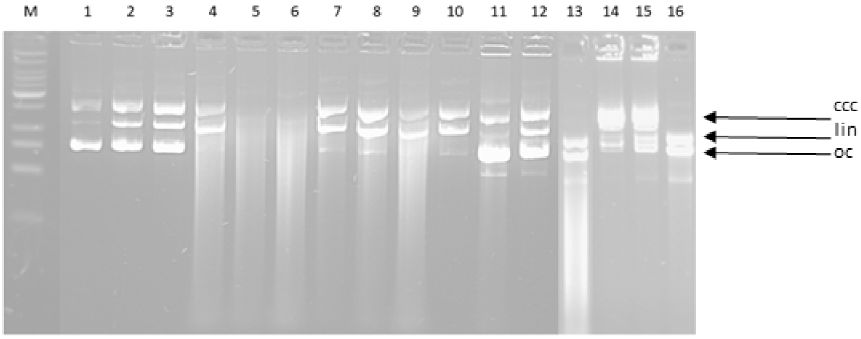

Figure S35. An example of an agarose gel electrophoresis separation of isolated plasmids DNA on R4 strains modified with selected coumarin with the formyl group at position C8 in its structure as carbonyl partners in a three-component Passerini reaction derivatives (lanes 1-16), and digested with repair Fpg protein. M = marker.

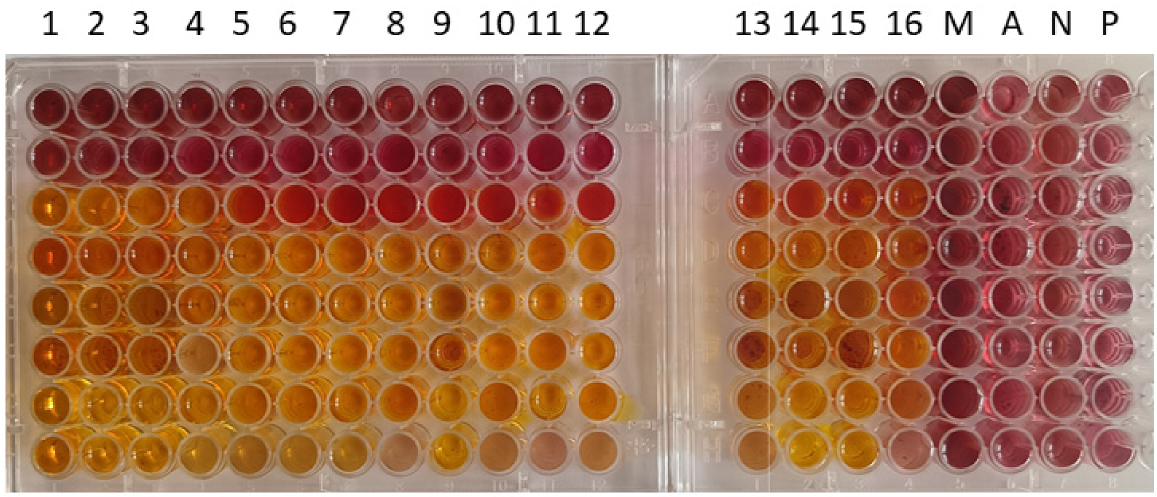

Figure S36. Configuration of the 96-well flat bottom plates in which the cytotoxicity assay was carried out on mouse embryonic fibroblast cell line BALB/c3T3**.** M= complete medium without cells. A= complete medium without extract solutions. N=negative control P=positive control Lanes from 1-16 tested compounds .

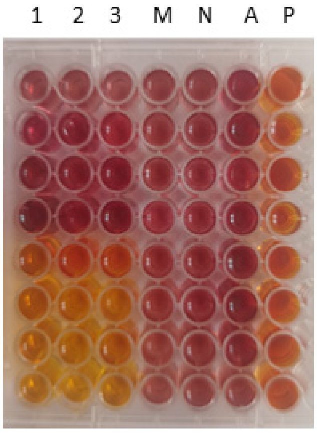

Figure S37. Configuration of the 96-well flat bottom plates in which the cytotoxicity assay was carried out on mouse embryonic fibroblast cell line BALB/c3T3**.** M= complete medium without cells. A= complete medium without extract solutions. N=negative control P=positive control Lanes from 1-3 tested compounds . 1-ciprofloxacin (cipro), 2-bleomycin (bleo), 3-cloxacillin (clox).

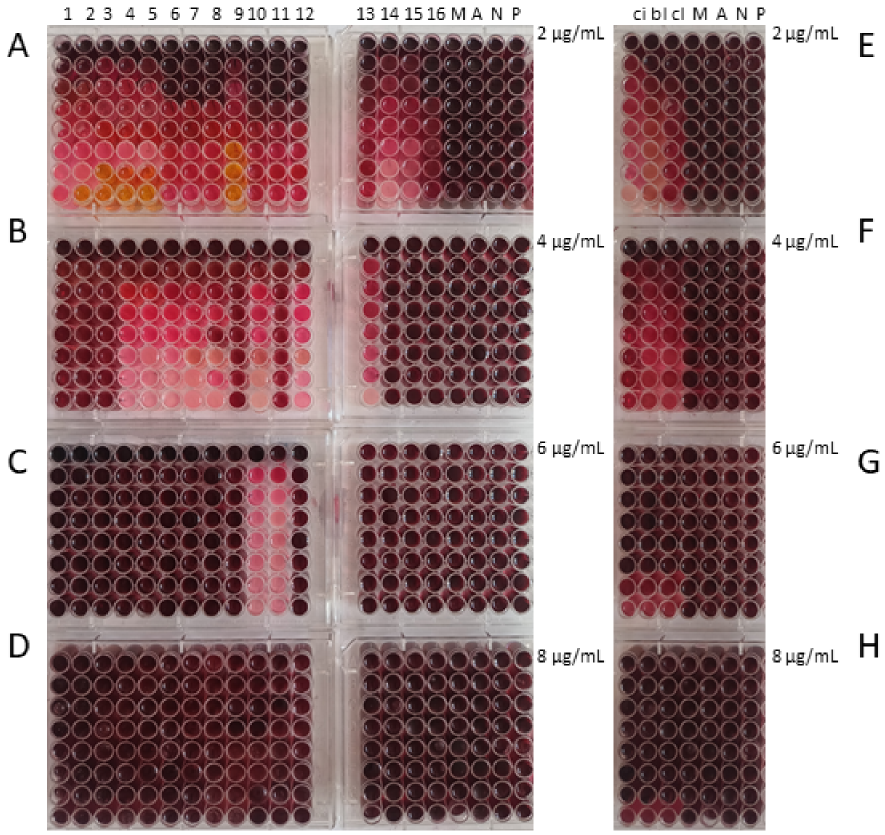

Figure S38. Configuration of the 96-well flat bottom plates in which the cytotoxicity assay was carried out on mouse embryonic fibroblast cell line BALB/c3T3. M= complete medium without cells. Panel A and E– 2 ug/ml concentration, Panel B and F- 4 ug/ml concentration, Panel C and G 6 ug/ml concentration, Panel D and H - 8 ug/ml concentration A= complete medium without extract solutions. N=negative control P=positive control. Lanes from 1-16 tested compounds . Lanes ci-ciprofloxacin, bl-bleomycin , cl- cloxacillin.
